# Multiparametric Ultrasonographic Phenotyping of the Masseter Muscle in Temporomandibular Disorders and Clinically Defined Probable Bruxism

**DOI:** 10.1111/joor.70262

**Published:** 2026-07-09

**Authors:** Alperen Tekin, Belde Arsan Özcan, Burcu Sümer, Büşra Erdoğan

**Affiliations:** ^1^ Department of Oral and Maxillofacial Radiology Faculty of Dentistry, Istanbul Medeniyet University Istanbul Türkiye

**Keywords:** bruxism, fractal analysis, masseter muscle, strain elastography, temporomandibular disorders, ultrasonography

## Abstract

**Background:**

Temporomandibular disorders (TMDs) and bruxism‐related masticatory muscle activity may influence masseter morphology, mechanical response and B‐mode texture, but their combined ultrasonographic phenotype is unclear.

**Objective:**

To evaluate whether clinically defined TMD status and probable bruxism‐positive phenotype are associated with multiparametric masseter ultrasound profiles.

**Methods:**

This prospective single‐centre study included 108 adults classified as TMD‐negative/bruxism‐negative controls (C0, *n* = 31), TMD‐negative/probable bruxism‐positive (T3, *n* = 25), TMD‐positive/bruxism‐negative (T2, *n* = 25) and TMD‐positive/probable bruxism‐positive (T1, *n* = 27). Sleep and awake bruxism were not separately distinguished. Bilateral thickness, cross‐sectional‐area‐derived sonographic estimated volume, strain elastography and resting fractal dimension (FD) were analysed offline by a single observer blinded to group allocation.

**Results:**

After false‐discovery‐rate correction, four‐group differences remained significant for resting/clenching thickness, estimated volume, strain elastography and resting FD (all *q* ≤ 0.001), with medium‐to‐large effects. T3 showed higher morphometric values, but sex‐stratified and sex‐adjusted analyses indicated that this finding required caution because of male predominance. GEE models identified sex, age, functional condition and group‐by‐condition terms as important determinants. FD repeatability was excellent (ICC [A,1] = 0.964; 95% CI, 0.941–0.977; coefficient of variation, 0.54%).

**Conclusion:**

Multiparametric masseter ultrasonography may capture complementary morphology, strain‐response and B‐mode texture phenotypes in TMD and clinically defined probable bruxism. Findings should be interpreted as phenotype‐enrichment rather than diagnostic evidence because TMD subtypes were heterogeneous and sleep/awake bruxism was not separately quantified.

## Graphical abstract

Multiparametric masseter ultrasonography combines B‐mode morphometry, CSA‐derived sonographic estimated volume, strain elastography and fractal‐dimension texture analysis to characterise masseter muscle phenotypes in adults classified by temporomandibular disorder status and clinically defined probable bruxism‐positive phenotype. The findings support phenotype enrichment rather than diagnostic classification and should be interpreted with consideration of TMD heterogeneity, sex distribution and the lack of separate sleep/awake bruxism quantification.

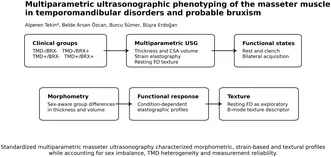

## 1 Introduction

Temporomandibular disorders (TMDs) comprise heterogeneous musculoskeletal and neuromuscular conditions involving the temporomandibular joints, masticatory muscles and associated structures. Their clinical manifestations may include pain, joint sounds, mandibular movement limitations and functional impairment, but these signs arise from different underlying phenotypes, including myogenous, joint‐related and mixed presentations [1, 2, 3, 4, 5]. This heterogeneity is clinically important because binary classification of TMD as present or absent can obscure distinct biomechanical and pain‐related mechanisms. Bruxism is also increasingly understood as a masticatory muscle activity or behaviour rather than a disease entity in itself. The international consensus distinguishes sleep bruxism and awake bruxism and recommends graded assessment approaches ranging from possible to probable and definite bruxism [6, 7]. In the present study, sleep and awake bruxism were not separately or instrumentally quantified; therefore, the bruxism variable is interpreted as a clinically defined probable bruxism‐positive phenotype. This terminology is used throughout the manuscript to avoid implying a direct disease effect of bruxism.

The relationship between TMD and bruxism remains clinically relevant but complex. A recent meta‐regression estimated a global co‐occurrence of bruxism and TMD of ~17% and reported that TMD prevalence among individuals with bruxism was ~63.5%, while also showing that demographic factors, particularly female proportion, may influence co‐occurrence estimates [8]. These data support simultaneous evaluation of TMD and bruxism while also emphasising the need for sex‐aware statistical interpretation. The masseter muscle is a logical target for this analysis. Repetitive jaw‐muscle activity may be associated with adaptive loading responses, whereas TMD‐related pain, altered motor control, protective inhibition and joint‐related dysfunction may modify muscle recruitment and mechanical behaviour. For this reason, the combined TMD/probable‐bruxism phenotype may be nonadditive rather than simply cumulative.

Ultrasonography is nonionising, accessible and suitable for repeated superficial muscle assessment. Standardised B‐mode imaging provides masseter thickness and CSA‐derived geometric information [9, 10, 11, 12, 13, 14, 15, 16, 17], whereas elastography and texture analysis may add complementary information on tissue deformation and greyscale patterning [18, 19, 20, 21, 22, 23, 24, 25, 26]. However, these methods require cautious interpretation. Strain elastography is semiquantitative and operator‐dependent, and the elastography ratio used here represents relative strain‐based behaviour rather than absolute tissue stiffness. Likewise, FD derived from B‐mode images reflects an image‐based texture descriptor and should not be treated as a direct histological biomarker of muscle microarchitecture without independent validation [22, 23, 24].

Therefore, this study aimed to evaluate the masseter muscle using a multiparametric ultrasound protocol in adults classified by TMD status and probable bruxism‐positive phenotype. The analytical framework explicitly incorporated TMD heterogeneity, sex imbalance, multiple comparisons, effect sizes and FD repeatability. We hypothesised that masseter morphology, CSA‐derived estimated volume, strain response and resting B‐mode texture would differ across clinically defined TMD/bruxism groups and that some findings would show nonadditive group‐by‐condition patterns.

## 2 Materials and Methods

### 2.1 Study Design and Ethical Approval

This prospective, single‐centre observational study was conducted at the Department of Oral and Maxillofacial Radiology, Faculty of Dentistry, Istanbul Medeniyet University, Türkiye. The protocol was approved by the Istanbul Medeniyet University Non‐Interventional Clinical Research Ethics Committee (ethics approval no. 2026‐GOSEK‐0129), and all participants provided written informed consent. The study is reported in accordance with observational research principles.

The primary imaging outcomes were bilateral masseter thickness, CSA‐derived sonographic estimated volume, strain elastography and rest‐state FD. Thickness, estimated volume and strain elastography were assessed at rest and during maximum voluntary clenching; FD was restricted to standardised resting B‐mode images.

### 2.2 Participants

Adults aged 18 years or older were recruited consecutively from faculty clinics. Participants were eligible if they could cooperate with clinical and ultrasonographic assessment and had complete group allocation and masseter ultrasound measurements. Exclusion criteria were factors that could independently alter masseter morphology or function, including major systemic or neuromuscular disease, previous facial trauma or surgery, botulinum toxin injection to the masticatory muscles within 6 months, current orthodontic treatment, pregnancy, intensive resistance training, metabolic disorders affecting muscle structure and medications potentially influencing muscle tone.

### 2.3 Clinical Assessment and Group Allocation

Each participant completed a structured symptom assessment and underwent a standardised clinical examination based on the DC/TMD framework [3, 4]. TMD status was assigned as present or absent for the primary factorial analysis. To address TMD heterogeneity, TMD‐positive participants were additionally characterised descriptively according to the predominant clinical subtype as myogenous‐predominant, joint‐related or mixed TMD. Participants were categorised as joint‐related when the clinical record indicated a dominant temporomandibular joint‐related diagnosis, as myogenous‐predominant when muscle pain was the predominant clinical presentation without a dominant joint‐related diagnosis and as mixed when both muscle and joint‐related components were recorded. The affected side was recorded as right, left or bilateral in TMD‐positive participants.

Bruxism status was assigned clinically as absent or probable bruxism‐positive based on the combination of reported parafunctional jaw‐muscle activity and compatible clinical indicators recorded during the standardised examination. This operational definition was intended to represent a clinically based probable bruxism‐positive phenotype rather than a definitive instrumental diagnosis. Sleep bruxism and awake bruxism were not separately distinguished, and no polysomnography, electromyography or ecological momentary assessment was performed. Therefore, all interpretations refer to a clinically defined probable bruxism‐positive phenotype rather than definite sleep or awake bruxism.

Final group allocation was based on the combination of TMD status and probable bruxism‐positive phenotype: C0, TMD‐negative/bruxism‐negative control; T3, TMD‐negative/probable bruxism‐positive; T2, TMD‐positive/bruxism‐negative; and T1, TMD‐positive/probable bruxism‐positive.

### 2.4 Ultrasonographic Acquisition Protocol

All examinations were performed with an Esaote MyLab X6 ultrasound system and SL1543 linear probe. Participants were seated upright with the Frankfort horizontal plane parallel to the floor and the head in a neutral position. Both masseter muscles were evaluated in the same acquisition sequence at rest and during maximum voluntary clenching. The transducer was positioned over the central belly of the masseter and aligned consistently relative to the zygomatic arch‐mandibular angle axis, with the imaging plane adjusted perpendicular to the skin surface and standardised across sides and conditions. Excessive probe compression was avoided.

To standardise operator technique, all acquisitions followed a prespecified protocol covering patient positioning, anatomical landmarking, probe orientation, compression control and ROI placement. Because the study used a single‐operator acquisition and offline‐analysis workflow, interoperator reliability could not be assessed.

B‐mode and strain elastography images were obtained in the same session. All ultrasound images were coded before offline analysis, and the observer performing measurements, ROI delineation and image analysis was blinded to group allocation.

### 2.5 Masseter Thickness Assessment

Masseter muscle thickness was assessed bilaterally under resting conditions and during maximum voluntary clenching. Participants were seated with the head in a neutral position, and a stable mandibular posture was maintained before each acquisition. The transducer was positioned transversely over the central belly of the masseter muscle, approximately at the region of greatest muscle convexity, and oriented perpendicular to the skin surface to reduce anisotropy and oblique measurement error. Excessive probe compression was avoided throughout image acquisition.

For each side and functional condition, the superficial and deep borders of the masseter were identified on B‐mode images, excluding the overlying fascia and the mandibular cortical shadow from the measurement line. Muscle thickness was measured as the shortest perpendicular distance between the superficial and deep muscle boundaries at the standardised mid‐belly region. Three repeated measurements were obtained for each side and condition, and the mean value was used for statistical analysis.

### 2.6 CSA‐Derived Sonographic Estimated Volume

CSA‐derived sonographic estimated volume was calculated separately for the right and left masseter muscles under resting and clenching conditions. First, the superoinferior extent of the muscle was assessed in the longitudinal plane. Because the complete length of the masseter could not consistently be included within a single sonographic field of view, consecutive longitudinal acquisitions were obtained when necessary. The inferior end of each acquisition was marked on the skin and used as the reference point for the subsequent acquisition, allowing continuous assessment from the superior to the inferior muscle border. Total muscle length was determined by summing the recorded longitudinal segments.

**FIGURE 1:**
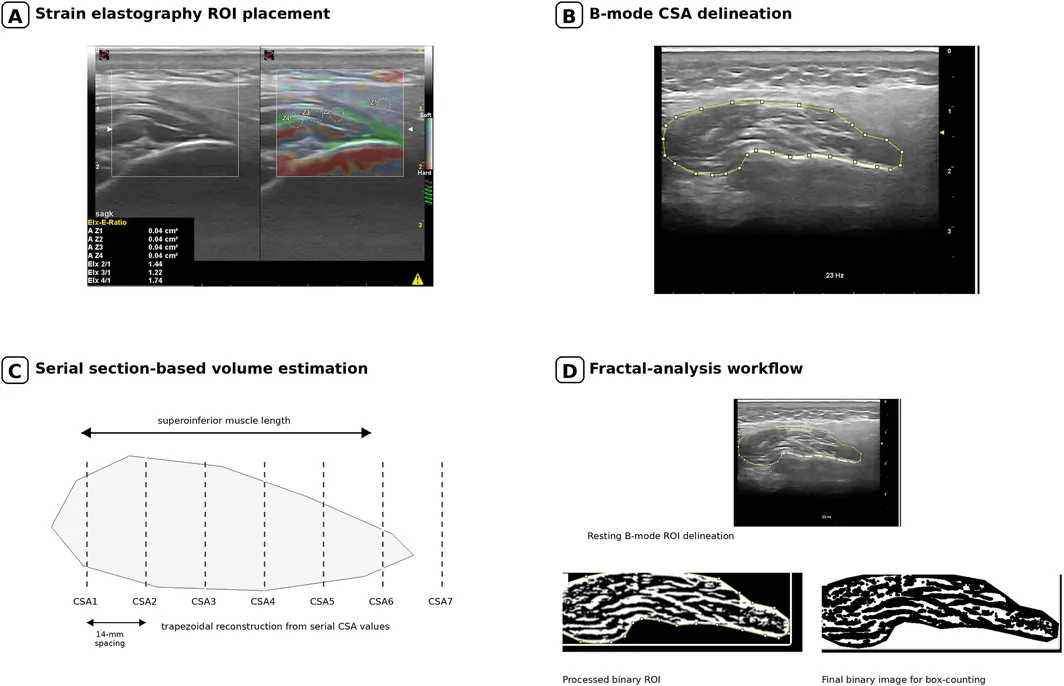
Representative multiparametric ultrasound and image‐processing workflow. (A) Strain elastography image showing ROI placement and ratio calculation. One reference ROI was placed within the ipsilateral parotid gland and three ROIs were placed within the masseter at comparable depths. (B) B‐mode image with polygon‐based masseter cross‐sectional area delineation. (C) Schematic illustration of serial section‐based CSA‐derived estimated volume reconstruction using consecutive transverse sections along the superoinferior muscle axis. (D) Fractal‐analysis workflow from resting B‐mode ROI delineation to the final binary image used for box‐counting FD calculation.

After longitudinal length assessment, serial transverse B‐mode images were acquired along the same superoinferior axis. Section levels were standardised according to the 14‐mm probe footprint. After each transverse acquisition, the inferior edge of the probe position was marked on the skin and used as the reference for the next section. When the remaining terminal segment was shorter than the full probe footprint, the actual remaining distance was recorded. For each transverse section, the visible masseter cross‐sectional area was manually delineated in Fiji/ImageJ using polygon‐based tracing while excluding the fascia, mandibular bone shadow and adjacent soft tissues. Representative CSA delineation and the serial section‐based volume reconstruction strategy are shown in Figure 1B,C.

Estimated volume was calculated using the trapezoidal rule, also referred to as the Average End Area Method, a serial‐section approach previously described for volume estimation, engineering volume calculation and ultrasonographic masseter volume assessment [27, 28, 29, 30], according to the following formula: V = Σ [(CSA*ᵢ* + CSA*ᵢ*₊₁) / 2 × L*ᵢ*], where CSA*ᵢ* and CSA*ᵢ*₊₁ represent two consecutive cross‐sectional areas and L*ᵢ* represents the distance between those sections. Segmental volumes were summed to obtain the total CSA‐derived sonographic estimated volume for each side and condition. Volumes were converted to cm^3^ for reporting. Because this method is based on serial two‐dimensional sonographic sections rather than direct three‐dimensional imaging, the derived value was interpreted as an estimated volume rather than as a reference‐standard anatomical volume.

### 2.7 Strain Elastography

Strain elastography was performed bilaterally under resting conditions and during maximum voluntary clenching using the same ultrasound system and transducer. The probe was positioned to include both the masseter muscle and the adjacent parotid gland within the same elastographic field whenever possible. Because strain elastography provides relative, semiquantitative information rather than absolute stiffness values, the ipsilateral parotid gland was used as the reference soft tissue.

For each side and condition, one circular ROI was placed within the parotid gland and three circular ROIs were placed within the masseter muscle. Each ROI was standardised to 0.04 cm^2^. The masseter ROIs were positioned within the visible muscle belly at comparable depths and away from fascia, bone shadow and image artefacts. Light rhythmic compression was applied with attention to the device's real‐time compression‐quality indicator, and measurements were accepted only when stable compression quality was achieved. Representative ROI placement and ratio calculation are shown in Figure 1A.

The device generated three masseter‐to‐parotid strain ratios for each side and condition. The mean of these three ratios was calculated. For analytical consistency with the directionality of the present dataset, the reciprocal of the mean strain ratio was calculated and used as the elastography value in all statistical analyses. Therefore, higher values indicate a greater relative strain‐based elastographic response within the context of this protocol. These values should not be interpreted as absolute tissue stiffness or directly compared with shear‐wave elastography values expressed in kPa.

### 2.8 Fractal Analysis

Fractal analysis was performed on standardised resting B‐mode ultrasound images of the masseter muscle. Image acquisition was performed using the same ultrasound system, linear transducer, patient position, anatomical region and acquisition protocol. To reduce technical variability, excessive probe compression and postacquisition image manipulation were avoided.

For each image, the masseter region of interest was manually delineated within the visible muscle borders, excluding the fascia, mandibular bone shadow and adjacent soft tissues. All selected ROIs were processed in Fiji/ImageJ using the same semiautomated macro and identical preprocessing workflow. After clearing the area outside the selected ROI, images were converted to 8‐bit greyscale. Background correction, contrast‐limited adaptive histogram equalisation, greyscale normalisation, and image rescaling were then applied to reduce low‐frequency greyscale inhomogeneity and improve texture‐pattern consistency across ROIs. Local thresholding with the Niblack method was used as an intermediate processing step, and final binary images for box‐counting analysis were generated using Otsu thresholding. The same preprocessing and thresholding sequence was applied uniformly to all ROIs.

Fractal dimension was calculated from the final binary images using the box‐counting method in the BoneJ plugin, with identical plugin settings applied to all images. The ROI delineation and binary‐image workflow used for FD calculation is summarised in Figure 1D. FD was interpreted as an exploratory descriptor of B‐mode ultrasound image texture complexity rather than as a direct histological biomarker of muscle structure.

### 2.9 Repeatability Assessment

Intraobserver repeatability of resting masseter FD was assessed in a randomly selected subset of 23 participants, including 46 right and left resting masseter B‐mode images. The same observer repeated FD measurements after a 2‐week interval. Before the second measurement session, images were coded and reordered and the observer was blinded to the initial measurements and clinical group allocation. Reliability was quantified using the intraclass correlation coefficient [ICC(A,1), two‐way mixed‐effects, absolute agreement, single measurement] with bootstrapped 95% confidence intervals. Agreement was additionally evaluated using Bland–Altman mean difference, 95% limits of agreement, repeatability coefficient, mean absolute difference and coefficient of variation.

### 2.10 Sample Size Considerations

The sample size was based on the available prospectively recruited cohort rather than on a predefined power calculation. The recruitment target was based on the four‐cell TMD/bruxism structure, repeated bilateral rest/clench measurements and the practical need to include at least 25 participants per analytical group. This is acknowledged as a limitation. The final sample size should be interpreted as suitable for detecting moderate‐to‐large group differences and interaction patterns, whereas smaller subgroup and sex‐stratified effects should be considered exploratory.

### 2.11 Statistical Analysis

Statistical analyses were performed using Python‐based scripts with SciPy and statsmodels. Categorical variables were summarised as counts and percentages. Continuous variables were summarised as mean ± SD when describing the sample and as median [interquartile range] for nonparametric group comparisons. Participant‐level bilateral means were used for primary four‐group descriptive comparisons.

Four‐group comparisons used Kruskal–Wallis tests, with epsilon‐squared reported as the global effect size. Pairwise post hoc comparisons used Mann–Whitney *U* tests with Holm adjustment and rank‐biserial correlation as the pairwise effect size. Rest‐to‐clench change was evaluated with Wilcoxon signed‐rank tests and matched‐pairs rank‐biserial correlation. Spearman correlations were used for rest‐state associations. False‐discovery‐rate correction was applied within predefined analytical families: global four‐group tests across imaging outcomes, sex‐stratified global tests, GEE coefficients within each outcome model, group‐by‐sex sensitivity terms and rest‐state correlation analyses. Pairwise post hoc comparisons were Holm‐adjusted within each outcome/condition family.

Adjusted repeated‐measures analyses used Gaussian generalised estimating equations with participant‐level clustering. Models included group, condition, side, sex, age and group‐by‐condition terms for outcomes assessed at rest and clench. Resting FD models included group, side, sex and age. Because reviewer concerns specifically addressed sex imbalance, additional sex‐stratified descriptive analyses and group‐by‐sex interaction models were performed. Global Kruskal–Wallis effect sizes were reported as epsilon‐squared and interpreted using conventional benchmarks of ~0.01, 0.06 and 0.14 for small, medium and large effects, respectively. For *r*‐type metrics, values of ~0.20, 0.40 and 0.70 were interpreted as small, medium and large effects, respectively, according to dentistry‐specific guidance [31]. GEE coefficients were interpreted as adjusted model‐based differences in the original outcome units.

## 3 Results

### 3.1 Study Population and Baseline Characteristics

A total of 108 participants were included: C0 (*n* = 31), T3 (*n* = 25), T2 (*n* = 25) and T1 (*n* = 27). Mean age was similar across groups (35.4–37.6 years). Sex distribution differed descriptively across groups: the T3 group included a higher male proportion (17/25, 68.0%), whereas C0 and T1 were predominantly female (23/31, 74.2%; and 21/27, 77.8%, respectively). This imbalance was explicitly addressed using sex‐stratified and sex‐adjusted analyses.

Among 52 TMD‐positive participants, the predominant clinical subtype was joint‐related in 35 participants, mixed in 10 participants and myogenous‐predominant in 7 participants. The affected side was bilateral in 22, left‐sided in 17 and right‐sided in 13 TMD‐positive participants. Baseline characteristics are summarised in Table 1. Additional distributions of dominant TMD subtype and affected side are provided in Tables S1 and S2.

**TABLE 1:** Baseline demographic and clinical characteristics of the four study groups.

Group label	*n* participants	Female *n* (%)	Male *n* (%)	Age, mean ± SD	Bruxism status	Dominant TMD subtype	Affected side
TMD−/BRX− control	31	23 (74.2)	8 (25.8)	36.4 ± 11.0	Negative	No TMD	Not applicable
TMD−/BRX+	25	8 (32.0)	17 (68.0)	37.6 ± 8.4	Probable bruxism‐positive phenotype	No TMD	Not applicable
TMD+/BRX−	25	14 (56.0)	11 (44.0)	35.4 ± 15.1	Negative	Joint‐related 19; mixed 2; myogenous‐predominant 4	Right 6; left 10; bilateral 9
TMD+/BRX+	27	21 (77.8)	6 (22.2)	35.5 ± 7.9	Probable bruxism‐positive phenotype	Joint‐related 16; mixed 8; myogenous‐predominant 3	Right 7; left 7; bilateral 13

*Note:* Values are shown as *n*, *n* (%) or Mean ± SD. Bruxism‐positive status denotes a clinically defined probable bruxism‐positive phenotype; sleep and awake bruxism were not separately distinguished. TMD subtype and affected side were recorded only for TMD‐positive participants.

### 3.2 Descriptive Ultrasonographic Outcomes With Multiple‐Comparison Control

**FIGURE 2:**
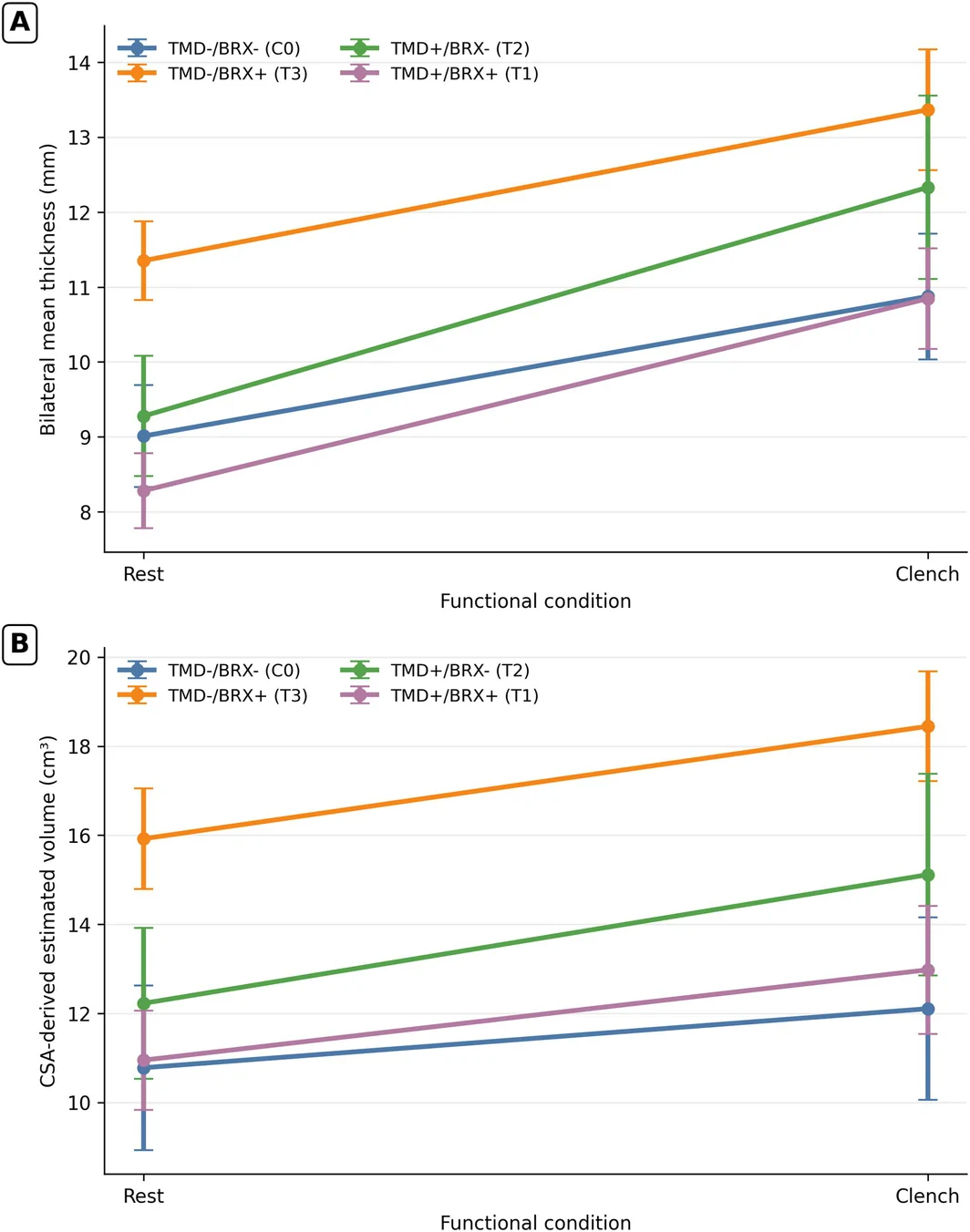
Rest‐to‐clench morphometric profiles by TMD/bruxism group. (A) Bilateral mean masseter thickness. (B) CSA‐derived sonographic estimated volume. Points and error bars represent mean values and 95% confidence intervals based on participant‐level bilateral means. These descriptive plots should be interpreted with the sex‐stratified and sex‐adjusted analyses reported in the Results.

Four‐group comparisons remained significant after false‐discovery‐rate correction for all primary imaging outcomes (Table 2). Resting thickness differed among groups (*q* < 0.001; epsilon‐squared = 0.313, large), as did clenching thickness (*q* < 0.001; epsilon‐squared = 0.178, large). Resting and clenching CSA‐derived estimated volume also differed among groups (both *q* < 0.001; epsilon‐squared = 0.228 and 0.223, respectively; large). The rest‐to‐clench morphometric profiles are shown in Figure 2.

**TABLE 2:** Bilateral mean ultrasonographic outcomes across TMD/bruxism groups after multiple‐comparison control.

Outcome	Condition	C0 median [IQR]	T3 median [IQR]	T2 median [IQR]	T1 median [IQR]	Global *q*	Effect size
Thickness, mm	Rest	8.83 [7.83, 10.04]	11.70 [10.70, 12.18]	8.82 [8.16, 9.89]	8.07 [7.45, 9.12]	< 0.001	0.313 (large)
Thickness, mm	Clench	11.20 [9.32, 12.24]	13.92 [12.68, 14.70]	12.76 [10.17, 12.91]	10.84 [10.11, 11.84]	< 0.001	0.178 (large)
CSA‐derived estimated volume, cm^3^	Rest	9.67 [8.10, 12.49]	16.52 [15.06, 17.53]	12.53 [9.81, 15.16]	10.52 [8.49, 13.06]	< 0.001	0.228 (large)
CSA‐derived estimated volume, cm^3^	Clench	12.02 [8.81, 14.61]	19.32 [16.93, 20.49]	15.00 [12.02, 18.93]	11.79 [10.52, 15.08]	< 0.001	0.223 (large)
Strain elastography value	Rest	0.89 [0.70, 1.12]	0.85 [0.62, 1.21]	1.15 [0.89, 2.22]	1.06 [0.97, 1.16]	0.001	0.125 (medium)
Strain elastography value	Clench	1.34 [1.08, 1.51]	1.46 [1.27, 1.88]	1.68 [1.41, 2.08]	1.85 [1.61, 2.17]	< 0.001	0.163 (large)
Fractal dimension	Rest	1.6343 [1.6236, 1.6423]	1.6553 [1.6350, 1.6898]	1.5737 [1.5630, 1.5860]	1.6415 [1.6262, 1.6637]	< 0.001	0.430 (large)

*Note:* Values are median [IQR] based on participant‐level bilateral means. Overall *q* values were obtained using false‐discovery‐rate correction of Kruskal–Wallis tests. Effect size is epsilon‐squared. CSA‐derived estimated volume is a standardised sonographic estimate, not reference‐standard MRI/CT volume.

**FIGURE 3:**
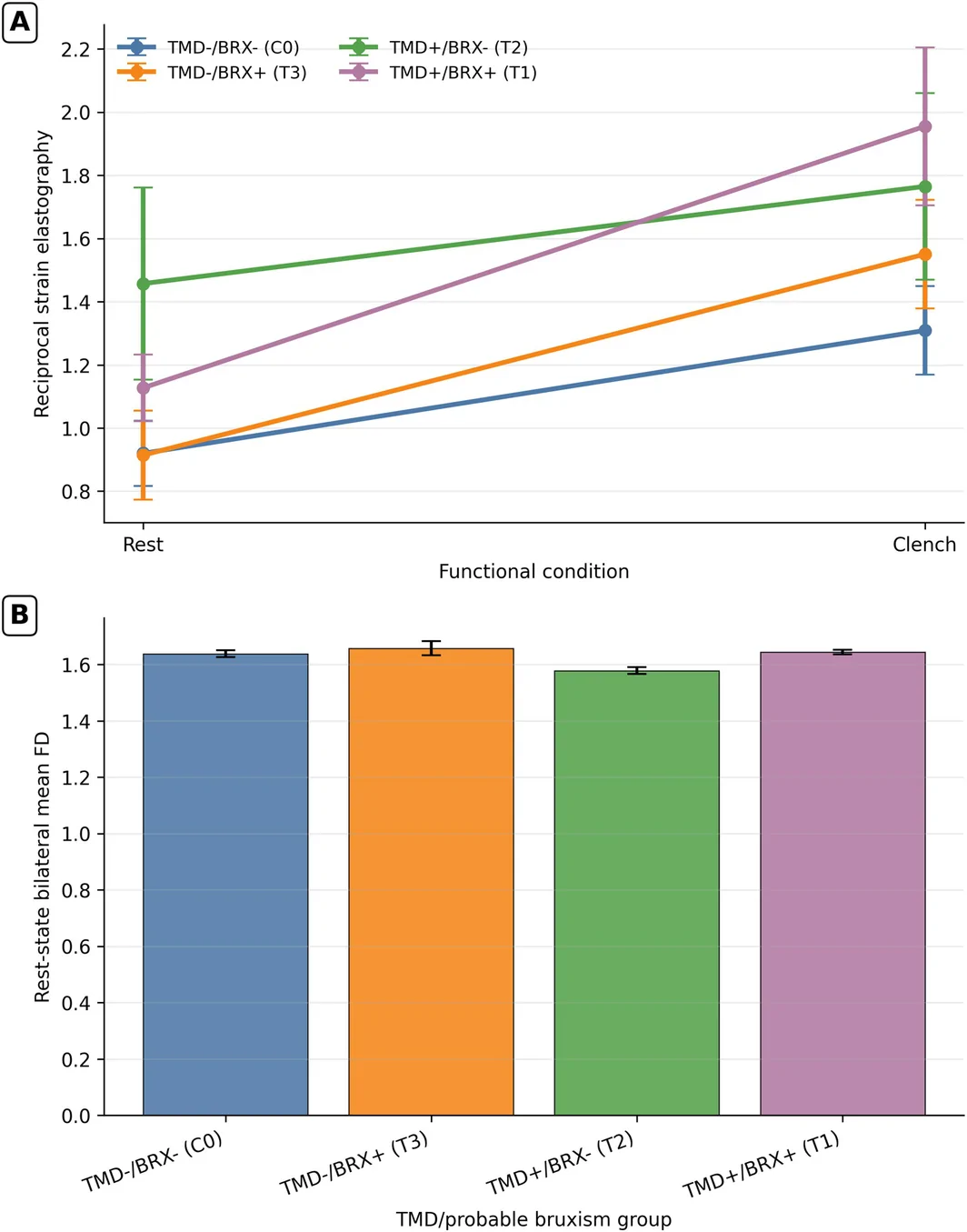
Elastographic and textural profiles by TMD/bruxism group. (A) Rest‐to‐clench elastography values. (B) Rest‐state bilateral mean FD. Points/bars and error bars represent mean values and 95% confidence intervals. FD is interpreted as an exploratory B‐mode texture descriptor.

Elastography values showed significant four‐group differences at rest (*q* = 0.001; epsilon‐squared = 0.125, medium) and during clenching (*q* < 0.001; epsilon‐squared = 0.163, large). Resting FD differed among groups with the largest global effect size among the nonparametric comparisons (*q* < 0.001; epsilon‐squared = 0.430, large). Elastographic and rest‐state FD profiles are shown in Figure 3.

Pairwise Holm‐adjusted analyses showed that the T3 group had higher morphometric values than C0 and T1 for thickness and estimated volume, particularly at rest and during clenching. However, because T3 contained a higher proportion of men, these morphometric patterns were not interpreted as bruxism‐specific effects without considering sex‐stratified and adjusted analyses.

### 3.3 Sex‐Stratified and Sex‐Adjusted Analyses

Sex‐stratified analyses were added because of the unequal sex distribution. In men, group differences remained evident for resting thickness (*q* = 0.035), resting and clenching elastography values (both *q* = 0.035) and resting FD (*q* = 0.002), whereas volume differences were not significant after correction. Among female participants, group differences remained significant for resting thickness, resting and clenching estimated volume, resting and clenching elastography values and resting FD after correction (all *q* ≤ 0.030).

In adjusted GEE models, female sex was associated with lower thickness (*β* = −1.19 mm; *q* = 0.002) and lower CSA‐derived estimated volume (*β* = −3.78 cm^3^; *q* < 0.001), confirming sex as an important morphometric determinant. The T3 group remained higher than C0 for thickness (*β* = 1.92 mm; *q* < 0.001) and estimated volume (*β* = 3.75 cm^3^; *q* < 0.001) after adjustment for sex and age, but these findings were interpreted cautiously because of the unequal group composition. Group‐by‐sex interaction terms did not remain significant after false‐discovery‐rate correction, including the T3‐by‐female term for estimated volume (*p* = 0.037; *q* = 0.110).

### 3.4 Adjusted Repeated‐Measures Models

Functional condition was a consistent determinant of thickness, CSA‐derived estimated volume, and elastography values. Clenching was associated with higher thickness (*β* = 1.86 mm; *q* < 0.001), higher estimated volume (*β* = 1.32 cm^3^; *q* < 0.001) and higher elastography values (*β* = 0.39; *q* < 0.001).

Group‐by‐condition terms indicated that clench‐related responses differed by clinical phenotype. For thickness, T2 and T1 showed greater condition‐related increases than C0 (T2: *β* = 1.19 mm, *q* < 0.001; T1: *β* = 0.70 mm, *q* = 0.015). For estimated volume, T3 and T2 showed greater clench‐related increases than C0 after correction (T3: *β* = 1.20 cm^3^, *q* < 0.001; T2: *β* = 1.56 cm^3^, *q* < 0.001), whereas the T1 term was smaller and did not remain significant after correction (*q* = 0.052). For elastography values, T3 and T1 showed greater condition‐related increases than C0 (both *q* = 0.011), whereas T2 did not.

### 3.5 Rest‐to‐Clench Functional Response

Within‐group rest‐to‐clench changes were significant with large matched‐pairs rank‐biserial effect sizes for thickness and estimated volume in all four groups after Holm adjustment. Elastography values increased significantly in C0, T3 and T1 after correction, but not in T2 (Holm‐adjusted *p* = 0.069), suggesting that elastographic response may vary according to TMD phenotype.

### 3.6 Fractal Analysis and Repeatability

The FD reliability analysis demonstrated excellent intraobserver repeatability. Across 46 resting images, ICC(A,1) was 0.964 (95% CI, 0.941–0.977). Bland–Altman analysis showed a negligible mean difference of −0.0001, with 95% limits of agreement from −0.0252 to 0.0250. The repeatability coefficient was 0.0251, the mean absolute difference was 0.0098 and the coefficient of variation was 0.54%. Side‐specific ICC values were 0.973 for the right side and 0.955 for the left side.

In the adjusted resting FD model, T2 showed lower FD than C0 (*β* = −0.066; *q* < 0.001). Female sex and age were also associated with lower FD values after correction. Side was not significant after correction (left vs. right: *p* = 0.038; *q* = 0.056). Therefore, side‐related and affected‐side FD findings were retained as exploratory rather than primary evidence.

### 3.7 Exploratory Rest‐State Correlations

**FIGURE 4:**
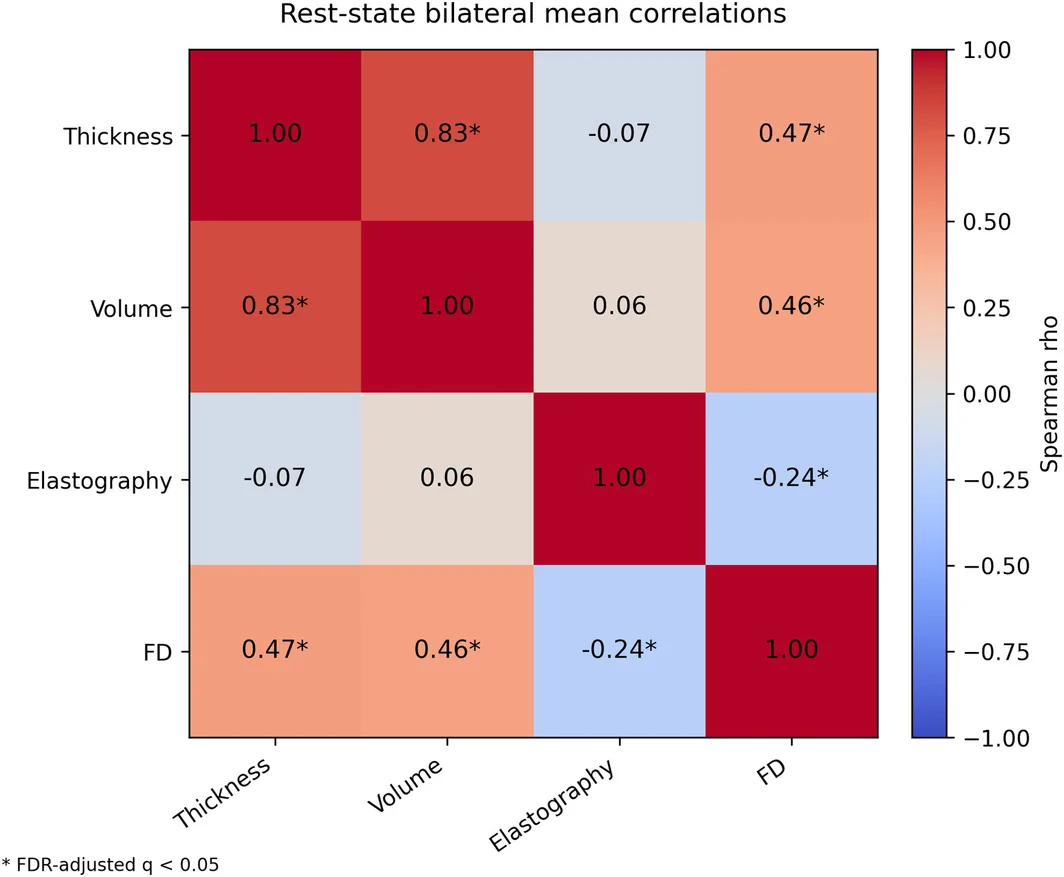
Exploratory Spearman correlation heatmap of rest‐state bilateral mean ultrasonographic variables. Correlation *p*‐values were corrected using false‐discovery‐rate procedures.

Rest‐state bilateral mean thickness and CSA‐derived estimated volume were strongly correlated (rho = 0.828; *q* < 0.001). Thickness and estimated volume were each moderately correlated with FD (rho = 0.475 and 0.458, respectively; both *q* < 0.001). Elastography values showed no meaningful correlation with thickness or estimated volume and a small negative correlation with FD (rho = −0.245; *q* = 0.016). The correlation pattern is summarised in Figure 4. Detailed pairwise post hoc comparisons, sex‐stratified analyses, adjusted GEE models, FD repeatability statistics, rest‐state correlations and effect‐size interpretation details are provided in Tables S3–S8.

## 4 Discussion

This analysis shows that clinically defined TMD status and probable bruxism‐positive phenotype are associated with distinct multiparametric masseter ultrasound profiles, although the findings should not be interpreted as a simple disease‐versus‐control comparison. After multiple‐comparison control and effect‐size reporting, global group differences remained evident for morphometric, elastographic and resting texture outcomes. However, the sex‐stratified and adjusted analyses demonstrate that group composition, especially the higher male proportion in the T3 group, is essential for interpreting morphometric differences.

The strongest morphometric pattern was the higher thickness and CSA‐derived estimated volume observed in the TMD‐negative/probable bruxism‐positive group. This finding is consistent with previous ultrasonographic work suggesting that bruxism‐related loading may be associated with increased masseter dimensions [32]. The pattern remained present in sex‐adjusted GEE models, but it should not be interpreted as a definitive effect of bruxism. Men generally have larger masticatory muscles, and the T3 group was male‐predominant. The group‐by‐sex interaction terms did not remain significant after correction, suggesting that sex did not fully explain the pattern. Therefore, we interpret this finding as a phenotype‐related association observed with consideration of sex distribution, rather than as evidence of a causal effect of bruxism.

The TMD‐positive groups showed more complex behaviour. In T2, resting FD was lower and elastography values were higher than in the control group in adjusted models, whereas T1 showed a greater clench‐related elastographic response. These findings may suggest that pain‐related or joint‐related TMD mechanisms influence masseter muscle recruitment and deformation differently from probable bruxism‐positive behaviour alone. This interpretation is consistent with the heterogeneous nature of TMD and with previous ultrasonographic and elastographic studies showing altered masseter morphology, echo characteristics and mechanical properties in TMD or myofascial pain populations [1, 2, 3, 4, 5, 14, 15, 16, 17, 18, 19, 20, 21, 33, 34]. This nonlinear pattern is compatible with the current view of bruxism as a behaviour that may act as a risk or protective factor depending on context [6, 7]. Because TMD‐positive participants included joint‐related, mixed and myogenous‐predominant presentations, the results should be considered phenotype‐generating rather than subtype‐specific.

The analysis also clarifies the interpretive status of FD. The excellent repeatability of FD supports the technical consistency of the measurement workflow. However, in B‐mode ultrasound, FD should be interpreted as an image‐derived texture descriptor that may reflect echo‐pattern complexity, rather than as a direct biomarker of muscle histology [22, 23, 24]. This distinction is important because FD is affected by acquisition settings, image preprocessing, thresholding and ROI placement. Accordingly, the lower resting FD observed in the TMD‐positive/probable bruxism‐negative group may suggest altered rest‐state masseter echotexture, but it should not be interpreted as evidence of a specific biological or histopathological mechanism. These findings indicate that FD may provide complementary quantitative information alongside conventional ultrasound and elastography, but they do not establish FD as a diagnostic marker of TMD or bruxism. This cautious interpretation is consistent with the broader ultrasound texture‐analysis literature, which suggests that B‐mode texture features may capture aspects of muscle quality but still require standardisation, diagnostic accuracy testing and validation against reference‐standard measures before being used as diagnostic biomarkers [26].

The reported volume values represent CSA‐derived sonographic estimates reconstructed from serial ultrasound sections using the trapezoidal/Average End Area approach [27, 28, 29, 30]. This approach may be useful for standardised within‐study phenotyping, but it should not be considered equivalent to reference‐standard anatomical volumetry.

The strain elastography findings should also be interpreted within their methodological context. The strain elastography ratio used in this study reflects relative tissue deformation compared with the parotid reference tissue, rather than absolute stiffness in kilopascals. Although strain elastography was selected because it was feasible with the available clinical ultrasound system and could be incorporated into a single‐session multiparametric protocol, it remains semiquantitative and operator‐dependent. Factors such as manual compression, transducer position, ROI placement, reference tissue selection and ratio definition may influence the measurements. Moreover, previous studies and reviews on masseter elastography in TMD and bruxism‐related conditions have used different elastographic methods and outcome metrics, limiting direct comparability [18, 19, 20, 21, 33]. Future studies using shear‐wave elastography may provide more reproducible and quantitatively comparable stiffness metrics.

The present study has several strengths. It used a four‐group factorial design, bilateral rest/clench acquisition, standardised single‐device imaging, coded blinded image analysis, repeatability assessment for FD and statistical reporting with multiple‐comparison control and effect sizes. These features improve transparency and reduce overinterpretation of isolated *p* values.

Important limitations remain. Sleep and awake bruxism were not separately distinguished, and no instrumental bruxism assessment was performed. Therefore, the bruxism‐positive group should be interpreted as a clinically defined probable phenotype. TMD was modelled primarily as a binary variable, even though descriptive subtype classification was added in the revision. The sample reflected the eligible cohort available during the study period, and sex distribution was uneven across groups. Although sex‐stratified and sex‐adjusted analyses were performed, residual confounding cannot be fully excluded. Formal repeatability was quantified for FD, but separate formal reliability estimates for thickness, CSA‐derived estimated volume and elastography values were not available. Finally, CSA‐derived estimated volume and FD require external validation before they can be used as stand‐alone clinical biomarkers.

Overall, these findings suggest that multiparametric masseter ultrasonography is more useful for phenotypic characterisation than for single‐variable diagnosis. Morphometric measures, strain‐based elastographic response and B‐mode texture appear to reflect partly different dimensions of masseter muscle behaviour. Considered together, these parameters may help generate testable hypotheses for future mechanistic and longitudinal studies on the interaction between TMD‐related mechanisms, clinically defined probable bruxism‐positive behaviour and masseter muscle adaptation.

## 5 Conclusion

In this prospective four‐group study, TMD status and clinically defined probable bruxism‐positive phenotype were associated with distinct and nonadditive multiparametric masseter ultrasound profiles. Following multiple‐comparison control, effect‐size reporting, sex‐stratified analyses and FD repeatability assessment, the findings support the use of masseter ultrasonography for phenotypic characterisation of the masseter muscle, rather than for direct diagnosis of TMD or bruxism. Future research should distinguish sleep and awake bruxism, include sex‐balanced samples, stratify TMD subtypes more comprehensively and validate CSA‐derived estimated volume and ultrasound FD against reference‐standard structural or functional measures.

**Bullet Points**

What is known: TMD and bruxism‐related masticatory muscle activity may influence the masticatory muscles, but previous ultrasound studies often focused on single parameters and did not fully address TMD heterogeneity or bruxism terminology.

What this study adds: A multiparametric ultrasound analysis showed corrected medium‐to‐large group differences in masseter morphology, relative strain behaviour and resting B‐mode texture while explicitly addressing sex imbalance, multiple comparisons, effect sizes and FD repeatability.

Clinical/research implication: Masseter ultrasound should be interpreted as a phenotype‐enrichment method, not as a stand‐alone diagnostic test for TMD or bruxism. Future studies should separate sleep and awake bruxism and examine TMD subtypes with balanced designs.

## Author Contributions

**Belde Arsan Özcan:** conceptualization, methodology, investigation, writing – review and editing, supervision. **Büşra Erdoğan:** investigation, writing – review and editing, data curation. **Burcu Sümer:** investigation, writing – review and editing, data curation. **Alperen Tekin:** conceptualization, methodology, investigation, data curation, formal analysis, writing – original draft, visualization, project administration.

## Funding

The authors have nothing to report.

## Ethics Statement

The study protocol was approved by the Istanbul Medeniyet University Non‐Interventional Clinical Research Ethics Committee (ethics approval number 2026‐GOSEK‐0129).

## Consent

Written informed consent was obtained from all participants.

## Conflicts of Interest

The authors declare no conflicts of interest.

## Supporting information


**Table S1:** Distribution of dominant TMD subtypes by analytical group.
**Table S2:** Affected‐side distribution by analytical group.
**Table S3:** Pairwise post hoc comparisons with Holm correction and rank‐biserial effect sizes. Outcome names are unchanged from the analysis dataset. Holm‐adjusted p values should be used for inference.
**Table S4:** Sex‐stratified four‐group comparisons.
**Table S5:** Adjusted GEE models. Models included group, condition, side, sex, age and group‐by‐condition terms for rest/clench outcomes. FD was modelled using resting side‐level data.
**Table S6:** Intraobserver reliability of resting masseter fractal dimension. FD measurements were repeated by the same observer two weeks later using coded and re‐ordered images.
**Table S7:** Rest‐state Spearman correlations with FDR correction.
**Table S8:** Effect‐size metrics and interpretation guide used in the revision.

## Data Availability

The data that support the findings of this study are available from the corresponding author upon reasonable request.
