# Supplementary material for: Multiparametric Ultrasonographic Phenotyping of the Masseter Muscle in Temporomandibular Disorders and Clinically Defined Probable Bruxism
Source: J Oral Rehabil. 2026 Jul 9;53(10):1960–71. doi: 10.1111/joor.70262 (PMC13583384; doi:10.1111/joor.70262)
Supplement: Supplementary file 1 [file JOOR-53-1960-s001.docx]

**Supplementary Material**

Tables are provided in a landscape layout for readability. Corrected P values should be used for inference.

**Supplementary Table S1. Distribution of dominant TMD subtypes by analytical group.**

| **Group** | **Group label** | **Dominant TMD subtype** | **n** |
| --- | --- | --- | --- |
| C0 | TMD−/BRX− control | No TMD | 31 |
| T3 | TMD−/BRX+ | No TMD | 25 |
| T2 | TMD+/BRX− | Joint-related | 19 |
| T2 | TMD+/BRX− | Mixed | 2 |
| T2 | TMD+/BRX− | Myogenous-predominant | 4 |
| T1 | TMD+/BRX+ | Joint-related | 16 |
| T1 | TMD+/BRX+ | Mixed | 8 |
| T1 | TMD+/BRX+ | Myogenous-predominant | 3 |

**Supplementary Table S2. Affected-side distribution by analytical group.**

| **Group** | **Group label** | **Affected side** | **n** |
| --- | --- | --- | --- |
| C0 | TMD−/BRX− control | Not applicable | 31 |
| T3 | TMD−/BRX+ | Not applicable | 25 |
| T2 | TMD+/BRX− | Right | 6 |
| T2 | TMD+/BRX− | Left | 10 |
| T2 | TMD+/BRX− | Bilateral | 9 |
| T1 | TMD+/BRX+ | Right | 7 |
| T1 | TMD+/BRX+ | Left | 7 |
| T1 | TMD+/BRX+ | Bilateral | 13 |

**Supplementary Table S3. Pairwise post hoc comparisons with Holm correction and rank-biserial effect sizes.**

*Outcome names are unchanged from the analysis dataset. Holm-adjusted P values should be used for inference.*

| **Outcome** | **Condition** | **Comparison** | **n1** | **n2** | Median difference | Raw P | Holm-adjusted P | Rank-biserial r | **\|r\| interpretation** |
| --- | --- | --- | --- | --- | --- | --- | --- | --- | --- |
| Thickness, mm | Rest | C0 vs T3 | 31 | 25 | -2.870 | <0.001 | <0.001 | -0.694 | medium |
| Thickness, mm | Rest | C0 vs T2 | 31 | 25 | 0.010 | 0.668 | 0.668 | -0.068 | very small/negligible |
| Thickness, mm | Rest | C0 vs T1 | 31 | 27 | 0.755 | 0.115 | 0.231 | 0.243 | small |
| Thickness, mm | Rest | T3 vs T2 | 25 | 25 | 2.880 | <0.001 | <0.001 | 0.629 | medium |
| Thickness, mm | Rest | T3 vs T1 | 25 | 27 | 3.625 | <0.001 | <0.001 | 0.893 | large |
| Thickness, mm | Rest | T2 vs T1 | 25 | 27 | 0.745 | 0.032 | 0.096 | 0.348 | small |
| Thickness, mm | Clench | C0 vs T3 | 31 | 25 | -2.721 | <0.001 | <0.001 | -0.599 | medium |
| Thickness, mm | Clench | C0 vs T2 | 31 | 25 | -1.566 | 0.075 | 0.163 | -0.280 | small |
| Thickness, mm | Clench | C0 vs T1 | 31 | 27 | 0.359 | 0.925 | 0.925 | 0.016 | very small/negligible |
| Thickness, mm | Clench | T3 vs T2 | 25 | 25 | 1.155 | 0.028 | 0.113 | 0.363 | small |
| Thickness, mm | Clench | T3 vs T1 | 25 | 27 | 3.080 | <0.001 | <0.001 | 0.662 | medium |
| Thickness, mm | Clench | T2 vs T1 | 25 | 27 | 1.925 | 0.054 | 0.163 | 0.313 | small |
| CSA-derived estimated volume, cm³ | Rest | C0 vs T3 | 31 | 25 | -6.856 | <0.001 | <0.001 | -0.621 | medium |
| CSA-derived estimated volume, cm³ | Rest | C0 vs T2 | 31 | 25 | -2.866 | 0.084 | 0.251 | -0.272 | small |
| CSA-derived estimated volume, cm³ | Rest | C0 vs T1 | 31 | 27 | -0.854 | 0.585 | 0.585 | -0.085 | very small/negligible |
| CSA-derived estimated volume, cm³ | Rest | T3 vs T2 | 25 | 25 | 3.990 | 0.002 | 0.007 | 0.517 | medium |
| CSA-derived estimated volume, cm³ | Rest | T3 vs T1 | 25 | 27 | 6.001 | <0.001 | <0.001 | 0.790 | large |
| CSA-derived estimated volume, cm³ | Rest | T2 vs T1 | 25 | 27 | 2.011 | 0.148 | 0.296 | 0.236 | small |
| CSA-derived estimated volume, cm³ | Clench | C0 vs T3 | 31 | 25 | -7.299 | <0.001 | <0.001 | -0.652 | medium |
| CSA-derived estimated volume, cm³ | Clench | C0 vs T2 | 31 | 25 | -2.985 | 0.035 | 0.105 | -0.332 | small |
| CSA-derived estimated volume, cm³ | Clench | C0 vs T1 | 31 | 27 | 0.223 | 0.400 | 0.400 | -0.130 | very small/negligible |
| CSA-derived estimated volume, cm³ | Clench | T3 vs T2 | 25 | 25 | 4.314 | 0.022 | 0.088 | 0.379 | small |
| CSA-derived estimated volume, cm³ | Clench | T3 vs T1 | 25 | 27 | 7.522 | <0.001 | <0.001 | 0.748 | large |
| CSA-derived estimated volume, cm³ | Clench | T2 vs T1 | 25 | 27 | 3.208 | 0.050 | 0.105 | 0.319 | small |
| Reciprocal strain elastography | Rest | C0 vs T3 | 31 | 25 | 0.041 | 0.575 | 0.575 | 0.089 | very small/negligible |
| Reciprocal strain elastography | Rest | C0 vs T2 | 31 | 25 | -0.259 | 0.002 | 0.011 | -0.489 | medium |
| Reciprocal strain elastography | Rest | C0 vs T1 | 31 | 27 | -0.167 | 0.008 | 0.031 | -0.410 | medium |
| Reciprocal strain elastography | Rest | T3 vs T2 | 25 | 25 | -0.299 | 0.004 | 0.018 | -0.482 | medium |
| Reciprocal strain elastography | Rest | T3 vs T1 | 25 | 27 | -0.208 | 0.029 | 0.088 | -0.354 | small |
| Reciprocal strain elastography | Rest | T2 vs T1 | 25 | 27 | 0.092 | 0.187 | 0.375 | 0.215 | small |
| Reciprocal strain elastography | Clench | C0 vs T3 | 31 | 25 | -0.112 | 0.047 | 0.141 | -0.312 | small |
| Reciprocal strain elastography | Clench | C0 vs T2 | 31 | 25 | -0.341 | 0.008 | 0.038 | -0.419 | medium |
| Reciprocal strain elastography | Clench | C0 vs T1 | 31 | 27 | -0.505 | <0.001 | <0.001 | -0.665 | medium |
| Reciprocal strain elastography | Clench | T3 vs T2 | 25 | 25 | -0.228 | 0.313 | 0.468 | -0.168 | very small/negligible |
| Reciprocal strain elastography | Clench | T3 vs T1 | 25 | 27 | -0.393 | 0.017 | 0.069 | -0.387 | small |
| Reciprocal strain elastography | Clench | T2 vs T1 | 25 | 27 | -0.164 | 0.234 | 0.468 | -0.194 | very small/negligible |
| Fractal dimension | Rest | C0 vs T3 | 31 | 25 | -0.021 | 0.008 | 0.025 | -0.414 | medium |
| Fractal dimension | Rest | C0 vs T2 | 31 | 25 | 0.061 | <0.001 | <0.001 | 0.854 | large |
| Fractal dimension | Rest | C0 vs T1 | 31 | 27 | -0.007 | 0.185 | 0.185 | -0.204 | small |
| Fractal dimension | Rest | T3 vs T2 | 25 | 25 | 0.082 | <0.001 | <0.001 | 0.862 | large |
| Fractal dimension | Rest | T3 vs T1 | 25 | 27 | 0.014 | 0.062 | 0.123 | 0.304 | small |
| Fractal dimension | Rest | T2 vs T1 | 25 | 27 | -0.068 | <0.001 | <0.001 | -0.883 | large |

**Supplementary Table S4. Sex-stratified four-group comparisons.**

| **Sex** | **Outcome** | **Condition** | **Group ns** | **H** | Raw P | epsilon-squared | **effect size interpretation** | FDR q |
| --- | --- | --- | --- | --- | --- | --- | --- | --- |
| Male | Thickness, mm | Rest | C0=8; T3=17; T2=11; T1=6 | 10.305 | 0.016 | 0.192 | large | 0.035 |
| Male | Thickness, mm | Clench | C0=8; T3=17; T2=11; T1=6 | 7.710 | 0.052 | 0.124 | medium | 0.073 |
| Male | CSA-derived estimated volume, cm³ | Rest | C0=8; T3=17; T2=11; T1=6 | 5.166 | 0.160 | 0.057 | small | 0.187 |
| Male | CSA-derived estimated volume, cm³ | Clench | C0=8; T3=17; T2=11; T1=6 | 1.690 | 0.639 | -0.034 | very small/negligible | 0.639 |
| Male | Reciprocal strain elastography | Rest | C0=8; T3=17; T2=11; T1=6 | 10.464 | 0.015 | 0.196 | large | 0.035 |
| Male | Reciprocal strain elastography | Clench | C0=8; T3=17; T2=11; T1=6 | 9.868 | 0.020 | 0.181 | large | 0.035 |
| Male | Fractal dimension | Rest | C0=8; T3=17; T2=11; T1=6 | 19.203 | <0.001 | 0.426 | large | 0.002 |
| Female | Thickness, mm | Rest | C0=23; T3=8; T2=14; T1=21 | 14.631 | 0.002 | 0.188 | large | 0.004 |
| Female | Thickness, mm | Clench | C0=23; T3=8; T2=14; T1=21 | 6.852 | 0.077 | 0.062 | medium | 0.077 |
| Female | CSA-derived estimated volume, cm³ | Rest | C0=23; T3=8; T2=14; T1=21 | 13.998 | 0.003 | 0.177 | large | 0.004 |
| Female | CSA-derived estimated volume, cm³ | Clench | C0=23; T3=8; T2=14; T1=21 | 18.160 | <0.001 | 0.245 | large | 0.001 |
| Female | Reciprocal strain elastography | Rest | C0=23; T3=8; T2=14; T1=21 | 9.279 | 0.026 | 0.101 | medium | 0.030 |
| Female | Reciprocal strain elastography | Clench | C0=23; T3=8; T2=14; T1=21 | 15.069 | 0.002 | 0.195 | large | 0.004 |
| Female | Fractal dimension | Rest | C0=23; T3=8; T2=14; T1=21 | 31.755 | <0.001 | 0.464 | large | <0.001 |

**Supplementary Table S5. Adjusted GEE models.**

*Models included group, condition, side, sex, age, and group-by-condition terms for rest/clench outcomes. FD was modelled using resting side-level data.*

| **Outcome** | **Term** | **Beta** | **SE** | **95% CI lower** | **95% CI upper** | P | FDR q |
| --- | --- | --- | --- | --- | --- | --- | --- |
| Thickness, mm | Group T3 | 1.915 | 0.407 | 1.117 | 2.714 | <0.001 | <0.001 |
| Thickness, mm | Group T2 | -0.019 | 0.390 | -0.783 | 0.745 | 0.962 | 0.962 |
| Thickness, mm | Group T1 | -0.748 | 0.358 | -1.449 | -0.047 | 0.037 | 0.046 |
| Thickness, mm | Clench vs Rest | 1.863 | 0.162 | 1.545 | 2.182 | <0.001 | <0.001 |
| Thickness, mm | Left vs Right | -0.252 | 0.103 | -0.453 | -0.051 | 0.014 | 0.020 |
| Thickness, mm | Female vs Male | -1.193 | 0.360 | -1.899 | -0.487 | <0.001 | 0.002 |
| Thickness, mm | Clench vs Rest | 0.150 | 0.297 | -0.433 | 0.733 | 0.614 | 0.683 |
| Thickness, mm | Clench vs Rest | 1.188 | 0.328 | 0.545 | 1.830 | <0.001 | <0.001 |
| Thickness, mm | Clench vs Rest | 0.699 | 0.267 | 0.176 | 1.222 | 0.009 | 0.015 |
| Thickness, mm | Age | -0.068 | 0.015 | -0.097 | -0.039 | <0.001 | <0.001 |
| CSA-derived estimated volume, cm³ | Group T3 | 3.745 | 0.915 | 1.951 | 5.539 | <0.001 | <0.001 |
| CSA-derived estimated volume, cm³ | Group T2 | 0.582 | 0.884 | -1.151 | 2.314 | 0.511 | 0.567 |
| CSA-derived estimated volume, cm³ | Group T1 | 0.148 | 0.792 | -1.404 | 1.700 | 0.851 | 0.851 |
| CSA-derived estimated volume, cm³ | Clench vs Rest | 1.325 | 0.157 | 1.017 | 1.633 | <0.001 | <0.001 |
| CSA-derived estimated volume, cm³ | Left vs Right | -0.573 | 0.316 | -1.192 | 0.046 | 0.070 | 0.087 |
| CSA-derived estimated volume, cm³ | Female vs Male | -3.784 | 0.700 | -5.155 | -2.413 | <0.001 | <0.001 |
| CSA-derived estimated volume, cm³ | Clench vs Rest | 1.198 | 0.315 | 0.580 | 1.816 | <0.001 | <0.001 |
| CSA-derived estimated volume, cm³ | Clench vs Rest | 1.561 | 0.408 | 0.762 | 2.360 | <0.001 | <0.001 |
| CSA-derived estimated volume, cm³ | Clench vs Rest | 0.702 | 0.336 | 0.044 | 1.360 | 0.036 | 0.052 |
| CSA-derived estimated volume, cm³ | Age | -0.174 | 0.032 | -0.237 | -0.112 | <0.001 | <0.001 |
| Reciprocal strain elastography | Group T3 | 0.009 | 0.092 | -0.172 | 0.189 | 0.926 | 0.926 |
| Reciprocal strain elastography | Group T2 | 0.532 | 0.160 | 0.218 | 0.846 | <0.001 | 0.005 |
| Reciprocal strain elastography | Group T1 | 0.200 | 0.066 | 0.071 | 0.329 | 0.002 | 0.008 |
| Reciprocal strain elastography | Clench vs Rest | 0.389 | 0.069 | 0.254 | 0.524 | <0.001 | <0.001 |
| Reciprocal strain elastography | Left vs Right | -0.048 | 0.061 | -0.167 | 0.072 | 0.434 | 0.620 |
| Reciprocal strain elastography | Female vs Male | 0.013 | 0.085 | -0.153 | 0.179 | 0.877 | 0.926 |
| Reciprocal strain elastography | Clench vs Rest | 0.247 | 0.089 | 0.073 | 0.421 | 0.005 | 0.011 |
| Reciprocal strain elastography | Clench vs Rest | -0.081 | 0.158 | -0.392 | 0.229 | 0.608 | 0.760 |
| Reciprocal strain elastography | Clench vs Rest | 0.439 | 0.158 | 0.130 | 0.748 | 0.005 | 0.011 |
| Reciprocal strain elastography | Age | -0.008 | 0.003 | -0.014 | -0.001 | 0.016 | 0.027 |
| Fractal dimension | Group T3 | 0.008 | 0.015 | -0.020 | 0.037 | 0.568 | 0.568 |
| Fractal dimension | Group T2 | -0.066 | 0.007 | -0.079 | -0.052 | <0.001 | <0.001 |
| Fractal dimension | Group T1 | 0.006 | 0.006 | -0.006 | 0.018 | 0.360 | 0.432 |
| Fractal dimension | Left vs Right | -0.007 | 0.004 | -0.014 | 0.000 | 0.038 | 0.056 |
| Fractal dimension | Female vs Male | -0.027 | 0.009 | -0.045 | -0.009 | 0.003 | 0.006 |
| Fractal dimension | Age | -0.001 | 0.000 | -0.001 | 0.000 | 0.001 | 0.004 |

**Supplementary Table S6. Intraobserver reliability of resting masseter fractal dimension.**

*FD measurements were repeated by the same observer two weeks later using coded and re-ordered images.*

| **Metric** | **n images** | **ICC(A,1)** | **95% CI** | **Mean difference** | **95% limits of agreement** | **Coefficient of variation** |
| --- | --- | --- | --- | --- | --- | --- |
| Overall FD | 46 | 0.964 | 0.941-0.977 | 0.000 | -0.0252 to 0.0250 | 0.54% |
| Right-side FD | 23 | 0.973 | 0.937-0.988 | 0.000 | -0.0223 to 0.0215 | 0.47% |
| Left-side FD | 23 | 0.955 | 0.918-0.974 | 0.000 | -0.0282 to 0.0287 | 0.61% |

**Supplementary Table S7. Rest-state Spearman correlations with FDR correction.**

| **Variable 1** | **Variable 2** | **n** | **Spearman rho** | Raw P | **\|rho\| interpretation** | **FDR q** |
| --- | --- | --- | --- | --- | --- | --- |
| Thickness, mm | CSA-derived estimated volume, cm³ | 108 | 0.828 | <0.001 | large | <0.001 |
| Thickness, mm | Reciprocal strain elastography | 108 | -0.070 | 0.469 | very small/negligible | 0.563 |
| Thickness, mm | Fractal dimension | 108 | 0.475 | <0.001 | medium | <0.001 |
| CSA-derived estimated volume, cm³ | Reciprocal strain elastography | 108 | 0.056 | 0.568 | very small/negligible | 0.568 |
| CSA-derived estimated volume, cm³ | Fractal dimension | 108 | 0.458 | <0.001 | medium | <0.001 |
| Reciprocal strain elastography | Fractal dimension | 108 | -0.245 | 0.011 | small | 0.016 |

**Supplementary Table S8. Effect-size metrics and interpretation guide used in the revision.**

| **Metric** | **Used for** | **Interpretation thresholds** |
| --- | --- | --- |
| Kruskal-Wallis epsilon-squared | Global nonparametric group comparisons | 0.01 small, 0.06 medium, 0.14 large (reported cautiously) |
| Rank-biserial r | Pairwise Mann-Whitney comparisons | \|r\| 0.20 small, 0.40 medium, 0.70 large |
| Spearman rho | Correlation analyses | \|rho\| 0.20 small, 0.40 medium, 0.70 large |
| Matched-pairs rank-biserial r | Within-group rest-to-clench Wilcoxon analyses | \|r\| 0.20 small, 0.40 medium, 0.70 large |
| GEE beta coefficient | Adjusted models | Reported as model-based adjusted difference with 95% CI; clinical relevance interpreted with outcome units and effect size pattern |
